# Multidimensional impact of breast cancer screening: Results of the multicenter prospective optisoins01 study

**DOI:** 10.1371/journal.pone.0202385

**Published:** 2018-08-23

**Authors:** Amélie Cariou, Roman Rouzier, Sandrine Baffert, Anne-Laure Soilly, Delphine Hequet

**Affiliations:** 1 Department of Surgical Oncology, Institut Curie, Saint-Cloud, France; 2 Inserm U900 –Bioinformatics, Biostatistics, Epidemiology and Computational Systems, Cancer Biology, Institut Curie, Saint-Cloud, France; 3 Department of Health Economics, Institut Curie, Paris, France; 4 Health Economics, Bourgogne Franche-Comté University, EA 7467, Dijon, France; University of South Alabama Mitchell Cancer Institute, UNITED STATES

## Abstract

Breast cancer (BC) screening has been developed to detect earlier stage tumors associated with better prognosis. The aim of study was to evaluate the impact of BC screening on therapeutic management of patients with first operable BC, and on costs, patients’ needs, and working life. OPTISOINS01 was a multicenter, prospective observational study which aimed to identify the main care pathway of early BC. Among patients aged from 50 to 74 years-old, 2 groups were defined: the “Clinical signs” group and the "Screening" group (national organized screening and individual screening). We compared between these 2 groups: locoregional and systemic treatments, direct medical and non-medical costs from a National Health Insurance perspective, patients’ needs assessed by the validated SCNS-BR8 “breast cancer” module of the SCNS-SF34 supportive care needs survey and the duration of sick leave. The “Clinical signs” group included 89 patients, while the”Screening” group included 290 patients. More axillary lymph node dissections and radical breast surgery were performed in the “Clinical signs”. The rate of adjuvant chemotherapy was dramatically higher in the “Clinical signs” group. The median direct medical costs of the “Screening” group were €11,860 (€3,643-€41,030) per year and per patient, much lower than in the “Clinical signs” group (€14,940; €5,317-€41,070). Finally, needs specifically assessed by the SCNS-BR8 questionnaire were significantly higher for the postoperative and post-adjuvant periods in the “Clinical signs” group. This study highlighted the benefit of BC screening in terms of reduced therapies and positive impact on work and social life.

## Introduction

Breast cancer (BC) remains the leading cause of cancer death among women worldwide [1]. When the tumor is detected and removed at an early stage (tumor size < 2 cm with no lymph node involvement), 5-year overall survival is greater than 90% [2]. BC screening has been developed to detect earlier stage tumors associated with better prognosis. In France, the organized BC screening program consists of mammography every 2 years between the ages of 50 and 74 years- and is free of charge for patients. However, the participation rate has been low and has remained stable since 2008, with regional disparities [3]. The national BC screening program is regularly criticized by certain health professionals, epidemiologists and the public, who denounce the risks of adverse effects, such as overdiagnosis and treatment of potentially non-aggressive tumors, or radiation-induced cancers and the lack of clear information given to women invited to participate in BC screening [4]. Data must be collected in order to clearly describe the pros and cons of BC screening. Although several studies have concluded on the benefit of BC screening in terms of overall survival [5, 6], few data are available concerning the impact of BC screening on treatments, costs and work and social life. The main objective of this prospective multicenter study was to evaluate the impact of BC screening on therapeutic management of patients with first operable BC. The secondary objectives were to assess the impact of BC screening on costs, patients’ needs, and working life.

## Patients and methods

OPTISOINS01 was a multicenter, prospective observational study conducted from December 2014 to March 2016 among BC patients from a regional health territory. The primary objective of the Optisoins01 study was to identify the main care pathway of early BC from diagnosis to 1-year follow-up, and to evaluate costs from various perspectives. The OPTISOINS01 study design has been previously described [7]. Eight nonprofit hospitals participated in the study: 3 teaching hospitals, 4 general hospitals and 1 comprehensive cancer center. Inclusion criteria were: women aged ≥18 years with previously untreated, first, histologically confirmed, operable BC. Exclusion criteria were: metastatic, locally advanced, or inflammatory BC, previous history of BC. The present study focused on patients aged 50 to 74 years, corresponding to the BC screening target population. Two groups were defined: the “Clinical signs” group and the "Screening" group (national organized screening and individual screening). Several factors were compared between these 2 groups: locoregional and systemic treatments, direct medical and non-medical costs from a National Health Insurance perspective, patients’ needs assessed by the validated SCNS-BR8 “breast cancer” module of the SCNS-SF34 supportive care needs survey [8] and the duration of sick leave. Fisher’s exact test or Student’s t-test were used to analyze these factors. Multivariate analysis was performed using a logistic regression model. Sick leave over a 1-year period was described according to whether or not the patients underwent BC screening. Differences in the areas under the curves of the 2 populations were compared to 1,000 permutations of random allocation of BC screening. Internal consistency and reproducibility of the SCNS-BR questionnaires were evaluated using Cronbach’s alpha coefficient. Analysis of factors associated with patient’s needs according to the 2 subgroups was carried out using the LOCF (Last Observation Carried Forward) method. The medical cost was assessed on the basis of National Health Insurance tariffs for consultations, examinations and medical procedures. We also evaluated the 1-year out-of-pocket health expenses including the costs associated with the use of health care services, alternative therapies, dietary supplements, specific cosmetic products, capillary pros- thesis, clothes, domestic help and travel expenses. These data were collected in a logbook filled out by patients throughout the year of follow-up. Differences were considered significant for p<0.05. All statistical analyses were performed with R software [9]. This study was registered with ClinicalTrials.gov (Identifier: NCT02813317) and was approved by the French National ethics committee (CCTIRS Authorization No. 14.602 and CNIL DR-2014-167) covering research at all participating hospitals.

## Results

### Population

Six hundred and four patients were included in the OPTISOINS01 study, 379 of whom were between the ages of 50 and 74 years. The “Clinical signs” group included 89 patients, while the”Screening” group included 290 patients. The participation rate in the organized screening program was 50%. Most BCs were invasive (n = 339) and about one-quarter of patients had lymph node involvement (n = 85). Nearly 40% of patients were employed (n = 154). The patient and cancer characteristics of the 2 groups were comparable except for lymph node involvement, which was observed more frequently in the “Clinical signs” group (34.8% (n = 31) vs 18.6% (n = 54), p<0.005).

### Locoregional and systemic treatments

More axillary lymph node dissections were performed in the “Clinical signs” group than in the “Screening” group (Table 1). In addition, patients in the “Screening” group were more frequently treated by conservative surgery, although this difference was not statistically significant. Outpatient surgery was performed more frequently in the “Screening” group. However. 3 determinants of outpatient surgery were identified on multivariate analysis: type of center (comprehensive cancer center; OR: 5.2; 95% CI: 3.1–8.7; p<0.005); type of breast surgery (conservative surgery; OR: 27.2; 95% CI: 13.6–58.6; p<0.005); and type of lymph node surgery (sentinel lymph node procedure; OR: 20.3; 95% CI: 6.1–85.6; p<0.005). The mode of cancer diagnosis was therefore not a determinant of outpatient surgery on multivariate analysis. Finally, the rate of adjuvant chemotherapy was dramatically higher in the “Clinical signs” group. In multivariate analysis, 3 factors were independently associated with adjuvant chemotherapy: age under 60 (OR: 5.6; 95% CI: 2.6–12.7; p<0.005), BC diagnosis on clinical signs (OR: 6.4; 95% CI: 1.8–31.2; p = 0.01) and axillary lymph node involvement (OR: 4.1; 95% CI: 1.9–9.2; p<0.005).

**Table 1:** Locoregional and systemic management in the “Screening” group and “Clinical signs” group.

	“Screening”, n = 290		“Clinical signs”, n = 89		P
	n or median	% or range	n or median	% or range	
					
**Surgery** Breast surgery					0.21
*Conservative*	242	83.4%	69	77.5%	
*Radical*	48	16.6%	20	22.5%	
Lymph node surgery					
*Sentinel lymph node*	216	74.5%	58	65.2%	**<0.005**
*Axillary clearance*	51	17.6%	30	33.7%	
*NA*	23	7.9%	1	1.1%	
Radiation therapy*					
*No*	35	12.1%	6	6.7%	0.25
*Breast*	253	87.2%	83	93.3%	
*Lymph nodes*	41	14.1%	25	28.1%	**0.01**
Chemotherapy					**<0.005**
*Yes*	83	28.6%	61	68.5%	
*No*	206	71.0%	28	31.5%	
*NA*	1	0.3%	0	0.0%	
Hormone therapy					0.33
*Yes*	209	72.1%	69	77.5%	
*No*	81	27.9%	20	22.5%	

*several responses

### Costs

#### Direct medical costs from a National Health Insurance perspective

**Fig 1:**
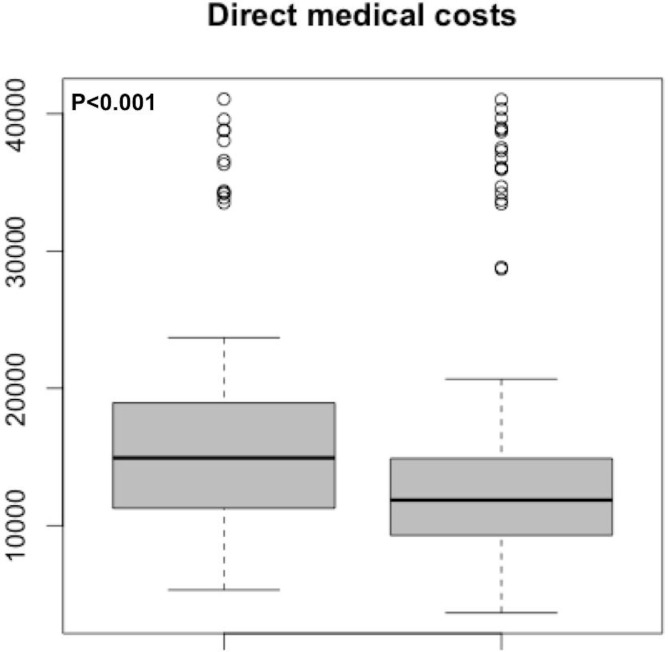
Median direct medical costs (€) per year and per patient in the “Clinical signs” group and the “Screening” group.

The median direct medical costs of the “Screening” group were €11,860 (€3,643-€41,030) per year and per patient (Fig 1), but remained much lower than in the “Clinical signs” group (€14,940; €5,317-€41,070, p<0.001).

#### Medical costs from a patient perspective

One-year out-of-pocket health expenses reached 614€ (10€ - 16 909€). No difference of cost was observed between the 2 groups.

### Work and social life

**Fig 2:**
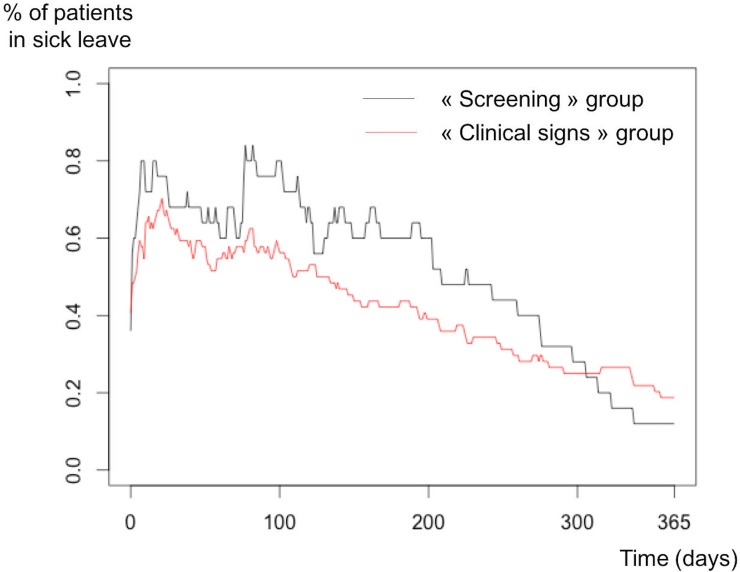
Duration of sick leave in the “Clinical signs” group and the “Screening” group.

One hundred fifty four (40.6%) patients of the study population were working at the time of diagnosis. A significant difference in terms of occupational categories was observed between the two groups, with more employees (37%, n = 41 vs 30%, n = 13, p = 0.01) and fewer white collar workers (29%, n = 32 vs 37%, n = 16, p = 0.01) in the “Screening” group. However, no difference in average income before treatment was observed between the 2 groups. No significant differences in wage changes and layoffs during treatment of the disease were observed between the 2 groups. On multivariate analysis, no factors were statistically associated with screening. A non-significant difference was observed for the duration of sick leave and the proportion of patients who stopped working for one year (Fig 2).

The SCNS-34 and SCNS-BR8 self-assessment questionnaires were distributed to all 604 patients in the OPTISOINS01 study. Despite the satisfactory response rate, an exhaustion effect was observed over time, with 82% of responses for the postoperative questionnaire, 70.7% of responses at the post-adjuvant period and 51% of responses during follow-up. An excellent internal consistency according to Cronbach's α coefficients was observed (Table 2).

**Table 2:** Internal validation of SNCS-34 and BR8 questionnaires.

	SNCS-34		BR-8	
	Cronbach’s α	95% CI	Cronbach’s α	95% CI
Postoperative	0.96	(0.95–0.97)	0.84	(0.79–0.87)
Post-adjuvant	0.97	(0.96–0.97)	0.81	(0.76–0.85)
Follow-up	0.97	(0.96–0.97)	0.84	(0.76–0.85)

**Fig 3:**
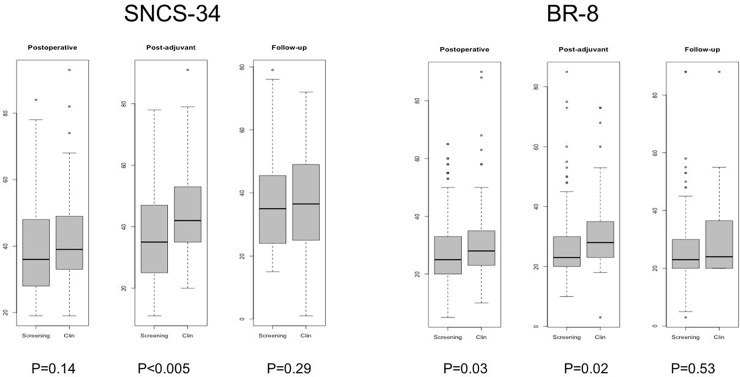
SNCS-34 and BR8 questionnaires at postoperative, post-adjuvant and follow-up times in the “Clinical signs” group and the “Screening” group.

Overall patients’ needs were estimated to be less than 50%. Analysis of the changes in patients’ needs over time showed stability with a slight tendency to decrease, except for the BR8 questionnaire, which showed a slight increase in needs during follow-up. Overall needs in this study population were significantly higher for the post-adjuvant period in the “Clinical signs” group (Fig 3) and needs specifically assessed by the SCNS-BR8 questionnaire were significantly higher for the postoperative and post-adjuvant periods in the “Clinical signs” group.

## Discussion

The OPTISOINS01 study explored multiple aspects of the impact of BC screening. Firstly, BC screening was associated with less aggressive therapies resulting in lower rates of axillary clearance and lymph node radiation. Although a difference in terms of conservative breast surgery rates was observed between the “Screening” group and the “Clinical signs” group, this difference was not statistically significant, which can be partly explained by the larger proportion of in situ carcinomas in the “Screening” group, that were more often locally extensive, and the high breast-conserving surgery rate in the overall population. However, the major result of this analysis concerns the decreased use of chemotherapy in the “Screening” group. To our knowledge, this is the first time that BC screening has been reported to have a major impact on systemic treatments. All these results indicate a higher proportion of earlier and less aggressive tumors in the “Screening” group. Patients diagnosed by screening have a better prognosis, as demonstrated in a cohort study including almost 1 million women between 1991 and 2005, with a 21% reduction of mortality in the screened population [10].

This study provides additional information on the impact of screening on health care costs and work and social life. Cancer has a major effect on work and difficulties are still described even 6 years after the end of treatment [11]. Women are traditionally more severely affected by these work difficulties than men, regardless of the cancer site [12]. We showed a non- significant trend for screening to reduce the prescription of sick leave in terms of both duration and number. This lack of statistical significance may be related to the small number of working patients (n = 156 working patients aged 50 to 74 in the study). However, patients’ needs were shown to be reduced in the “Screening” group, particularly during the adjuvant and postoperative periods. BC screening therefore meets the objectives of the 3^rd^ National Cancer Program, which particularly supports cancer education programs to improve quality of life [13].

In addition to the published benefits of BC screening in terms of survival, the positive impact in terms of treatment received and psychosocial impact highlighted in this study provide additional elements in support of BC screening. However, BC screening rates remain low, estimated, as only 51.5% of women were screened in 2015, while the target population represented 4.3 million women/year [14]. The current debate focuses on BC overdiagnosis (cancer that would not have been responsible for death or that would not have presented clinically during the woman's lifetime), secondary to screening consequently resulting in over-treatment. In 2015, the BC overdiagnosis rate was estimated to be 19% [6]. However, health authority recommendations support BC screening, which results in a reduction of BC mortality, outweighing the risks associated with irradiation and overdiagnosis [5, 6].

## Conclusion

This multicenter prospective study highlighted the benefit of BC screening in terms of reduced therapies and positive impact on work and social life. The major result concerns a dramatic reduction of chemotherapy in screened patients, resulting in reduced health costs and better quality of life. Despite the criticisms of BC screening, such as overdiagnosis or breast irradiation, health authorities support this national program. The results of this study provide additional arguments to encourage patients to participate in the screening program.
